# Multi-modal deep learning for intelligent landscape design generation: A novel CBS3-LandGen model

**DOI:** 10.1371/journal.pone.0328138

**Published:** 2025-07-24

**Authors:** Mingzhen Lu, Lili Shi

**Affiliations:** 1 School of Design Wenzhou, Wenzhou Polytechnic, Wenzhou, China; 2 School of Artificial Intelligence, Wenzhou Polytechnic, Wenzhou, China; Dr. NGP Institute of Technology, INDIA

## Abstract

With the acceleration of the global urbanization process, landscape design is facing increasingly complex challenges. Traditional manual design methods are gradually unable to meet the needs for efficiency, precision, and sustainability. To address this issue, this paper proposes an intelligent landscape design generation model based on multimodal deep learning, namely CBS3-LandGen. By integrating image data, text data, and generation optimization techniques, this model can generate landscape plans that meet the design objectives within limited time and resources.Specifically, the model employs the ConvNeXt network to process image data, uses the BART model to analyze text information, and optimizes the generation effect through StyleGAN3. This multimodal architecture enables the model to perform excellently in terms of image generation quality, text generation consistency, and the fusion of images and text. In the experiments, we trained and tested the model using the DeepGlobe and COCO datasets. The results show that on the DeepGlobe dataset, the Frechet Inception Distance (FID) is 25.5 and the Inception Score (IS) is 4.3; on the COCO dataset, the FID is 30.2 and the IS is 4.0. These results demonstrate the superiority of CBS3-LandGen in generation tasks, especially in aspects such as image quality, diversity, and multimodal data fusion. The method proposed in this paper provides new ideas for intelligent landscape design and promotes the integration of landscape design and deep learning technologies. Future research will further optimize the model’s performance, improve training efficiency, and expand its application potential in practical landscape design, urban planning, ecological protection, and other fields. The code for implementing CBS3-LandGen Model is available at https://github.com/LMZ81/CBS3-LandGen.git.

## Introduction

Landscape design, as an interdisciplinary creative activity, encompasses multiple aspects such as spatial planning, ecological design, functional layout, and aesthetic expression [1]. With the acceleration of globalization and urbanization, achieving efficient and sustainable landscape design within the constraints of limited land resources and complex ecological environments has become one of the core challenges in urban planning and environmental design [2]. However, traditional landscape design methods often rely on manual expertise and handcrafted operations, which are inefficient and prone to subjective biases from designers. These limitations fail to meet the modern requirements for diversity, innovation, and sustainability in urban landscape design [3]. Therefore, leveraging advanced technologies to assist or replace traditional design workflows has become an essential approach to enhancing the efficiency and quality of landscape design.

In recent years, deep learning has achieved remarkable breakthroughs in fields such as computer vision and natural language processing. Its powerful feature extraction and pattern recognition capabilities present new opportunities for landscape design [4]. Multimodal learning, in particular, has emerged as a method integrating different data sources—such as images, text, and geographic information—to address the challenges of processing and analyzing diverse datasets in landscape design [5]. Multimodal deep learning enables the fusion of information from various modalities, providing more comprehensive and accurate design solutions. However, landscape design involves multiple data modalities, including images, text, and geospatial information. The heterogeneity of these data poses significant technical challenges for deep learning in achieving effective integration [6]. Balancing design innovation with multiple constraints—such as aesthetics, functionality, and ecology—remains a pressing issue for generating landscape designs using deep learning.

Against this backdrop, this study proposes the CBS3-LandGen Model, an intelligent landscape design generation network based on multimodal deep learning. The model integrates image data (e.g., remote sensing images, topographic maps), textual data (e.g., design descriptions, functional requirements), and generation optimization techniques to achieve automated landscape design generation. It employs the ConvNeXt network to process image modalities, the BART model for textual understanding and generation, and StyleGAN3 to optimize the generation outputs. This modular and interpretable architecture enhances the degree of automation in landscape design and offers an intelligent solution for future urban landscape planning.

The contributions of this study are summarized as follows:

- The CBS3-LandGen model is proposed, which effectively combines image, text, and generative optimization modules through multimodal deep learning technology, providing an innovative generative framework for intelligent landscape design.
- The effective fusion of images and text is achieved, with the collaborative work of the ConvNeXt and BART models solving the fusion and generation issues of different modality data, offering more comprehensive and accurate design solutions for landscape design.
- The effectiveness and superiority of the model are verified through experiments, comparing it with existing methods, and demonstrating through ablation experiments and visual results the collaborative effect of each module and the application potential of the model in landscape design.

The following sections of this paper will introduce the related research background, provide a detailed description of the proposed model architecture, present the experimental process and results, and conclude with a summary and outlook for future research.

## Related works

### Deep learning in landscape design

Deep learning technologies have achieved significant breakthroughs across various domains, particularly in computer vision, natural language processing, and generative modeling. These rapid advancements present unprecedented opportunities for the field of landscape design [6,7]. Traditional landscape design methods often rely on designers’ expertise and manual drafting, making it difficult to efficiently handle complex spatial relationships and diverse design requirements. The introduction of deep learning, particularly models such as CNNs and GANs, enables the automatic extraction of features from data and the optimization of spatial and functional relationships, significantly improving design efficiency and quality [8]. For instance, recent deep learning-based methods for image recognition and generation can automatically identify different landform types, create diverse landscape renderings, and even optimize the layout of elements such as buildings and vegetation. However, the application of these technologies in landscape design remains in its infancy and faces numerous challenges [9].

Landscape design inherently exhibits strong multimodal characteristics, involving the integrated application of data in various forms such as images, text, and geographic information. While deep learning has achieved remarkable success in processing unimodal data, effectively integrating these heterogeneous data types poses a significant technical challenge [10]. For example, combining visual elements of landscape design (e.g., topographic maps and plant distribution) with textual information from design documents (e.g., functional requirements and ecological considerations) to generate practical and context-aware design solutions remains a key research difficulty [11]. In recent studies, deep feature extraction methods based on the attention mechanism have been proposed, demonstrating how to enhance the interpretability of models and their ability to handle complex inputs in multimodal learning [12,13]. In recent years, the interpretability technology based on attention mechanism has been widely studied, showing the potential to promote modality alignment and enhance model interpretation ability in multimodal generation tasks (for example, the relevant exploration of Dgxainet model in the field of medical imaging [14]), providing new ideas for solving this problem. To this end, this paper proposes an intelligent landscape design generation network based on multimodal deep learning. By effectively fusing image and text modalities, it aims to improve the automation and intelligence level of landscape design generation and overcome the technical challenges in multimodal data fusion.

### Multimodal learning and landscape design

Multimodal learning has emerged as a significant focus in recent deep learning research, enabling the processing and integration of information from diverse modalities, such as images, text, and audio. Traditional machine learning methods typically rely on a single data source for model training, which often falls short in providing sufficient contextual information when tackling complex problems [15,16]. In contrast, multimodal learning simultaneously processes multiple types of data, offering a more comprehensive understanding and modeling of real-world problems. This approach is particularly advantageous for tasks requiring the integration of rich contextual and spatial information [17–19]. In the field of landscape design, the multimodal nature of data is especially pronounced—image data provides visual insights into terrain, architecture, and vegetation, while textual data conveys design requirements, functional planning, and cultural context. Effectively integrating these heterogeneous data sources is a core challenge in multimodal learning [20].

However, in practical applications, multimodal data fusion is far from straightforward. Different modalities exhibit significant variations in data structures and representations. For instance, image data is typically represented as high-dimensional tensors, whereas text data exists as sequences or word embeddings. These differences create a technical challenge in achieving effective integration within a unified model. Furthermore, the relationships between modalities are often complex and nonlinear [21]. Extracting and leveraging these intermodal relationships to achieve optimal fusion performance remains an unsolved issue. Although strategies such as shared feature spaces and alignment-based learning have made progress, research on multimodal fusion is still in its exploratory phase [22,23]. This study combines image processing, text understanding, and generative optimization to provide an innovative solution for multimodal data fusion in landscape design. This approach aims to achieve more efficient and accurate design generation by overcoming existing technical barriers.

### Generative networks in landscape design

Generative networks, particularly Generative Adversarial Networks (GANs) and their variants, have become a pivotal research area in deep learning in recent years. These networks generate highly realistic images by training two opposing models—a generator and a discriminator—demonstrating powerful generative capabilities [24]. GANs have achieved remarkable success in computer vision applications, especially in tasks such as image generation, inpainting, and style transfer, where they produce visually compelling images that meet specific requirements [25,26]. In landscape design, generative networks have been widely employed for automated generation, optimization, and style transformation of landscape imagery. For example, GAN models can generate landscape images tailored to specific design needs, such as eco-friendliness or functional zoning [27,28]. By carefully controlling the generation process, these networks can optimize multiple design objectives, better meeting the constraints of complex design scenarios [29].

However, landscape design demands more than just generating visually harmonious images; it also requires consideration of multidimensional factors, such as spatial layout, functional zoning, and ecological balance. Existing generative networks often focus on single-objective optimization, such as image quality or style transformation, and lack the ability to comprehensively optimize for complex design goals [30,31]. Additionally, challenges such as image quality and stability persist in generative networks like GANs. In the design domain, generated images must not only exhibit aesthetic quality but also align with specific functional requirements and environmental conditions [32]. Thus, the integration of generative networks with diverse design constraints represents a critical challenge in landscape design generation.

In recent years, with the rapid development of multimodal learning, an increasing number of studies have begun to attempt to integrate data from different modalities, such as images and texts, into generative networks, thereby enhancing the capabilities of these networks [33]. In the field of landscape design, researchers have started to focus on how to combine data from different modalities (such as image data, design documents, functional requirements, etc.) to carry out more comprehensive design generation. For example, some works have introduced bimodal inputs of images and texts, enabling the generative network to generate landscape design plans that meet the requirements not only based on visual information but also according to textual descriptions [34]. Recent studies (such as VILBERT, CLIP, etc.) have demonstrated the great potential of the fusion of images and texts in generative tasks. Especially in complex design tasks, this multimodal input significantly improves the flexibility and accuracy of generation. However, multimodal data fusion has brought new challenges, especially in terms of how to effectively jointly model different modalities [35]. Research shows that how to enhance the synergistic effect among multimodal data without sacrificing the generation effect remains a difficult problem in the current research on landscape design generation.

To address this issue, this paper proposes using StyleGAN3 as a generative optimization module, combining multimodal data of images and texts to further improve the quality and stability of the landscape design generative network.

## Methods

### Overview of the model

**Fig 1:**
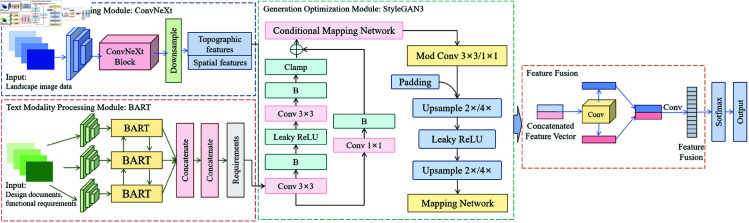
The core structure and data flow of the CBS3-LandGen Model. It consists of three main components: the image modality processing module (ConvNeXt), the text modality processing module (BART), and the generative optimization module (StyleGAN3). Each module processes specific types of input data sequentially, ultimately generating high-quality landscape design solutions that meet the desired objectives.

The CBS3-LandGen Model integrates multiple modules, including image processing, text processing, and generative optimization, to address the challenges of multimodal data fusion and generative optimization in landscape design. The input multimodal data includes landscape design image data (such as topographic maps, spatial layout diagrams), text data (such as design requirements, functional needs), and potential design constraint information. To efficiently process these heterogeneous data types, the model is divided into three main modules: the image modality processing module, the text modality processing module, and the generative optimization module. Fig 1 clearly illustrates the functional division of each module and the data flow process.

As shown in Fig 1, the image modality processing section employs the ConvNeXt network. The main function of this module is to extract deep features from the input image data, such as terrain variation, vegetation distribution, and building layout. This module processes the input landscape images using Convolutional Neural Networks (CNN), converting them into more abstract feature representations to facilitate subsequent design generation.

The text modality processing module uses the BART model, which is responsible for extracting key information from text data, such as design documents and functional requirements, and generating design objectives that are integrated with the image data. Understanding text data not only helps generate landscape designs that meet actual needs but also provides more explicit constraint conditions for the image generation process.

The generative optimization module utilizes StyleGAN3, and its task is to generate high-quality landscape images that align with the design objectives based on the features and requirements output by the previous two modules. By optimizing the generation process, StyleGAN3 can simultaneously consider aesthetic effects, functional layouts, and ecological adaptability during image generation, resulting in more complete and precise landscape design solutions.

The close collaboration of the three modules enables the CBS3-LandGen Model to efficiently fuse multimodal data and generate intelligent landscape designs, ensuring that the model can generate landscape solutions that meet actual needs based on input multimodal data. This improves the diversity and stability of the generated results, providing a more comprehensive solution for future intelligent landscape design.

### Image modality processing module: ConvNeXt

**Fig 2:**
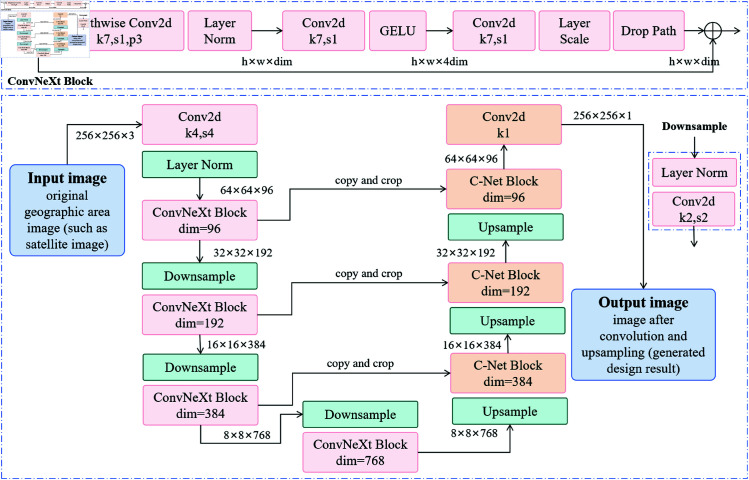
Image modality processing flow of the ConvNeXt module, showing the structure and data flow of the ConvNeXt network in image modality processing. This includes convolutional layers, activation functions, pooling layers, and fully connected layers, and how these layers extract advanced features and spatial information from the images.

In the CBS3-LandGen Model, the core task of the image modality processing module is to extract deep features from the input landscape images so that the subsequent design generation can be optimized based on the structure, texture, and spatial layout information of the images. In this module, we use the ConvNeXt network, an improved architecture based on CNN, which achieves efficient image feature extraction through deep convolutions and cross-layer connections [36,37]. ConvNeXt optimizes traditional CNNs by improving convolution operations and activation functions to enhance its ability to recognize complex image patterns [38]. Fig 2 shows the specific structure of this module, which includes multiple convolutional layers, activation layers, and pooling layers, ultimately generating high-dimensional feature representations of the images through a fully connected layer.

As shown in Fig 2, the input image first undergoes a series of convolutional layers for feature extraction. Let the input image be $I\in {\mathbb{R}}^{H\times W\times C}$, where *H* and *W* are the height and width of the image, and *C* is the number of channels. Through convolution operations, the spatial information and texture features of the image are gradually extracted and mapped into a high-dimensional space.

$$F_{\text{conv}}=\sigma (W_{\text{conv}}*I+b_{\text{conv}})$$ (1)

where $W_{\text{conv}}$ is the convolution kernel, * denotes the convolution operation, $b_{\text{conv}}$ is the bias term, and σ is the activation function. After several layers of convolution and pooling operations, the features of the image become increasingly abstract, and the final feature map $F_{\text{conv}}$ has higher spatial and semantic representational capabilities.

Furthermore, this work introduces cross-layer connections and deep convolutional structures into ConvNeXt, ensuring that the feature extraction process not only relies on shallow features but also effectively integrates deep information. After feature extraction by a series of convolution layers, the output feature vector $V_{\text{conv}}$ is sent to the fully connected layer for further processing. Let the output of the fully connected layer be $V_{\text{conv}}\in {\mathbb{R}}^{D}$, where *D* represents the feature dimension. The fully connected operation can be represented as:

$$V_{\text{conv}}=W_{\text{fc}}F_{\text{conv}}+b_{\text{fc}}$$ (2)

where $W_{\text{fc}}$ is the weight matrix of the fully connected layer and $b_{\text{fc}}$ is the bias term. At this point, the advanced features of the image have been effectively extracted and can provide rich semantic information for the subsequent design generation.

In the image modality processing, we not only extract basic visual features from the images but also model the spatial structure. For example, different regions in landscape design may have different functional and aesthetic requirements. By combining spatial information with image features, ConvNeXt can generate more accurate feature representations for each image region. Let $S\in {\mathbb{R}}^{N}$ represent the spatial information, where *N* is the number of spatial regions. We can combine the spatial information and image features in a weighted manner to obtain the final spatial-image fused feature $F_{\text{fusion}}$:

$$F_{\text{fusion}}=\sum_{i=1}^{N}\alpha_{i}F_{i}+\beta S_{i}$$ (3)

where $\alpha_{i}$ and β are the weighting coefficients, and *F*_*i*_ and *S*_*i*_ represent the features of the *i*-th image and spatial region, respectively. In this way, we can effectively combine the visual information of the image with the spatial layout, generating feature representations that better meet design requirements.

Finally, the feature representation $F_{\text{fusion}}$ generated by ConvNeXt is sent to subsequent modules for joint modeling with text data. Through this image modality processing and feature fusion, the CBS3-LandGen Model is able to capture the details and structural features of the image in landscape design, laying a foundation for generating landscape solutions that meet design needs.

The ConvNeXt module extracts high-level features from the images through deep convolutions and cross-layer connections, and combines spatial information for weighted fusion, providing rich visual input for landscape design generation. From image feature extraction to the final feature fusion, it ensures the efficient processing and application of image data in the model.

### Text modality processing module: BART

**Fig 3:**
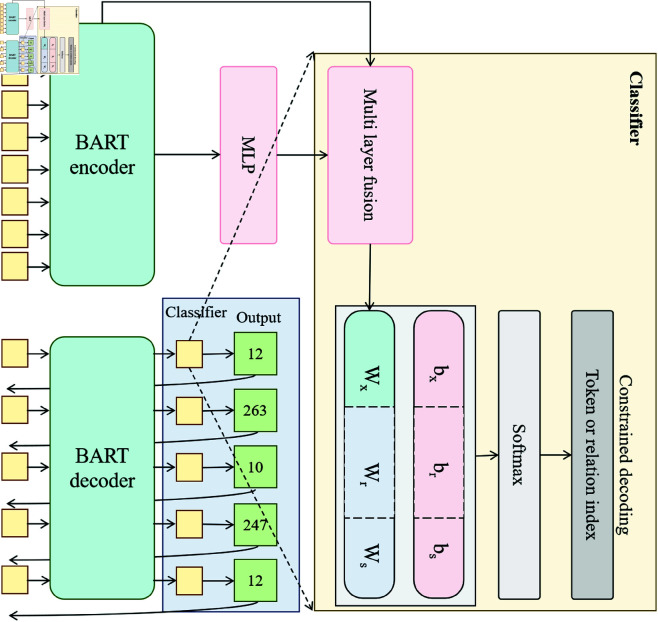
BART Text Modality Processing Flow: It shows the structure and data flow of the BART model in text modality processing, including text encoding, contextual modeling, and decoding processes, extracting and generating textual information that meets design objectives from design documents and requirements.

In the CBS3-LandGen Model, the core task of the text modality processing module is to extract key information from design documents and functional requirements and generate design objectives that align with image modality data. This module uses the BART model (Bidirectional and Auto-Regressive Transformers), which combines the advantages of bidirectional encoders and autoregressive decoders. It effectively processes contextual information in text and generates textual descriptions or design solutions that correspond to the input image [39,40]. Fig 3 shows the structure of this module, which includes the stages of text input encoding, contextual information learning, and text output generation. The application of this module in landscape design is mainly to extract useful information from textual data such as design requirements, functional needs, and ecological constraints, and by learning the relationships between this information and image data, it generates landscape solutions that meet design objectives.

As shown in Fig 3, the input text is first converted into a vector representation through an embedding layer. Let the input text be $T=[t_{1},t_{2},...,t_{n}]$, where *t*_*i*_ represents the *i*-th word in the text and *n* is the length of the text. The embedding layer transforms the text into a low-dimensional word vector representation $W_{T}\in {\mathbb{R}}^{n\times d}$, where *d* is the dimension of the word vectors. In this way, each word in the text is mapped to a continuous vector space, preserving its semantic information. Next, the text vector passes through the BART encoder, which uses a self-attention mechanism to model the context of the text [41]. The output of the encoder can be expressed as:

$$E_{T}=\text{Encoder}(W_{T})$$ (4)

where $E_{T}\in {\mathbb{R}}^{n\times d^{\prime }}$ is the encoded text representation, and $d^{\prime }$ is the feature dimension of the encoder output. At this point, the contextual information of the text has been effectively captured, providing strong support for subsequent design generation.

Next, the encoded output of the text is passed into the decoder, which generates new text outputs through an autoregressive mechanism. For example, in generating each word, the decoder predicts the next word based on the previously generated words and the encoder’s output. Let the current step be *t*_*k*_, the decoder’s output can be expressed as:

$$P(t_{k}|T)=\text{Decoder}(E_{T},[t_{1},...,t_{k-1}])$$ (5)

where *P*(*t*_*k*_|*T*) represents the probability of generating word *t*_*k*_ given the context *T*. Through this autoregressive generation process, the BART model is able to generate textual descriptions or design solutions that align with actual design requirements, based on the contextual information of the text.

In addition to the traditional autoregressive generation method, the BART model also employs a denoising autoencoder strategy. By masking part of the text information, the model can better learn long-range dependencies between words. During training, the model’s loss function can be expressed as:

$$L_{\text{BART}}=-\sum_{k=1}^{n}\log P(t_{k}|T)$$ (6)

where $L_{\text{BART}}$ is the training loss of the BART model, and the goal is to minimize the difference between the generated text and the real text. By optimizing this loss function, the BART model can continuously adjust its parameters to generate more accurate and design-targeted textual information.

Additionally, to ensure the effective fusion of text and image information, the CBS3-LandGen Model introduces a cross-modal correlation learning mechanism. Specifically, it establishes a shared feature space between the image and text modalities, allowing both to align at the same semantic level. Let the feature output from the image modality be $F_{\text{image}}$ and from the text modality be $F_{\text{text}}$. We optimize the following objective function to achieve feature alignment between them:

$$L_{\text{fusion}}=||F_{\text{image}}-F_{\text{text}}|{|}^{2}$$ (7)

where $L_{\text{fusion}}$ is the loss for cross-modal feature fusion, and the goal is to minimize the difference between the image and text modality features, ensuring that the model can learn in a common semantic space, generating more coordinated design solutions.

Finally, the BART text modality processing module, through bidirectional encoding and autoregressive decoding mechanisms, effectively learns the complex dependencies in text data, generating textual descriptions or design solutions closely related to image data and meeting landscape design requirements. These generated texts will work together with the outputs from the image modality to assist the entire system in the automated generation of high-quality, multi-objective optimized landscape designs.

### Generation optimization module: StyleGAN3

**Fig 4:**
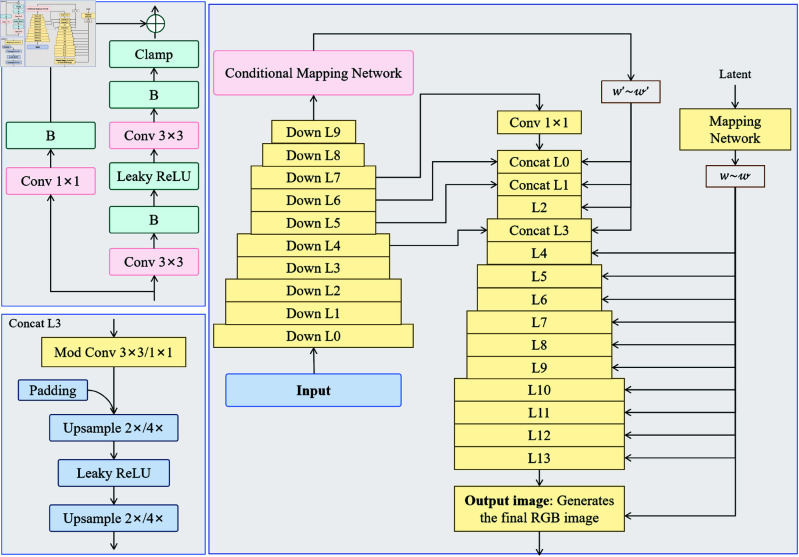
Structure and Process of the StyleGAN3 Generation Optimization Module. It shows the structure of StyleGAN3 in the generation optimization module, focusing on the relationship between the generator, discriminator, and latent space optimization, and how latent vectors and style controls are used to generate high-quality landscape design images that meet multimodal data needs.

In the CBS3-LandGen Model, the generation optimization module is a crucial component of the entire model. Its primary task is to generate high-quality images that meet landscape design goals based on the outputs of the first two modules (image modality processing and text modality processing). We adopt StyleGAN3 as the generation optimization module. It is the latest variant of GANs, known for its enhanced generation capabilities and stability [42]. StyleGAN3 can generate realistic images that meet complex constraints. Fig 4 shows the structure of StyleGAN3, including the generator, discriminator, and optimization of the latent space. The generator creates images from latent vectors, while the discriminator assesses whether the generated images meet design requirements. Through adversarial training, the generator’s performance is optimized. The advantage of StyleGAN3 lies in its fine control capabilities, enabling the generation of high-quality images and making adjustments in the latent space to ensure the generated images better align with the multimodal data design requirements [43,44].

As shown in Fig 4, the input latent vector $z\in {\mathbb{R}}^{m}$ is passed to the generator, which gradually generates the image through multiple convolutional layers and activation functions. The output of the generator can be expressed as:

G(𝐳)=Generator(𝐳) (8)

where $G(z)\in {\mathbb{R}}^{H\times W\times C}$ represents the generated landscape image, with *H* and *W* being the height and width of the image, and *C* being the number of channels. The generator performs optimization in the latent space *z* through multiple convolutional operations and style adjustment layers, ensuring that the generated image not only meets design requirements but also exhibits diversity and creativity.

StyleGAN3 adjusts the image generation process through style vectors, making the image generation more flexible. In the style adjustment layers, the generator transforms the latent vector to adjust the features of the image at different layers, such as texture, color, and shape. Let the style vector be $s\in {\mathbb{R}}^{d}$. We can control the generation process through the following formula:

$$\hat{G}(𝐳,𝐬)=G(𝐳)⊙𝐬$$ (9)

where ⊙ denotes the element-wise multiplication between the style vector and the image, with *s* controlling the features of each layer of the image, thus changing the style and content of the generated image. This style control mechanism allows StyleGAN3 to generate landscape design images that are personalized to different design needs while maintaining high image quality.

In addition to the image generation capability, the discriminator in StyleGAN3 also plays a key role. The discriminator’s task is to distinguish between generated images and real images, providing feedback to the generator to optimize the generation process. The output of the discriminator is *D*(*x*), representing the probability that the image *x* is a real image. The discriminator makes its judgment through the following formula:

$$D(𝐱)=\sigma (W_{D}\cdot 𝐱+b_{D})$$ (10)

where σ is the Sigmoid function, and *W*_*D*_ and *b*_*D*_ are the weights and bias of the discriminator, respectively. The generator and discriminator jointly optimize through adversarial training, improving the realism of the generated images and their ability to meet design requirements.

To achieve multi-objective optimization in landscape design, StyleGAN3 combines features from both image and text modalities. By optimizing the objective function, the generated image not only meets the visual requirements from the image data but also aligns with the functional requirements and design constraints expressed in the text data. Let the features from the image modality be $F_{\text{image}}$, and the features from the text modality be $F_{\text{text}}$. The quality of the generated image *G*(*z*) and the difference between the two modality features can be evaluated by the following objective function:

$$L_{\text{gen}}=||{𝐅}_{\text{image}}-{𝐅}_{\text{text}}|{|}^{2}+\lambda \cdot D(G(𝐳))$$ (11)

where $L_{\text{gen}}$ is the total loss for generation optimization, and λ is a balancing term that controls the weight between image quality and modality feature consistency. By minimizing this loss function, the generator can adjust its generation process to ensure that the final generated landscape design image has high visual quality and satisfies the functional requirements and design constraints.

Additionally, StyleGAN3 adopts a progressive generation strategy, generating the image layer by layer rather than generating the entire image at once. This ensures the stability and consistency of the generated image. This strategy helps to avoid unnatural textures or structural distortions during the generation process, enhancing the stability of the generated image. The generated image can be expressed as:

$$\hat{G}(𝐳,𝐬)=\sum_{i=1}^{n}\alpha_{i}\cdot G_{i}(𝐳,𝐬)$$ (12)

where *G*_*i*_(*z*,*s*) is the image generated at the *i*-th layer by the generator, and $\alpha_{i}$ is the weight coefficient for each layer. The complete landscape design image is obtained by weighted fusion of these layers.

Overall, the StyleGAN3 generation optimization module, through adjustments in the latent space, style control, discriminator optimization, and the design of the multi-objective loss function, generates images from the input latent vector to the output image. Through adversarial training and multimodal data fusion, the module ensures that the generated images are high-quality and meet multimodal data requirements, while also making the generation process more stable and controllable.

### User control mechanism

**Fig 5:**
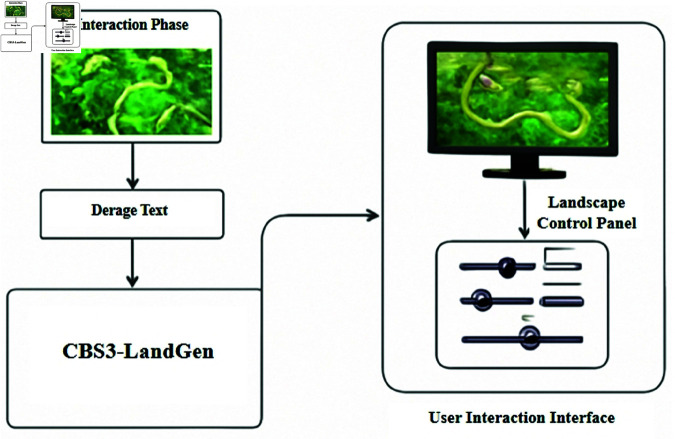
Schematic diagram of the user control mechanism of the CBS3-LandGen model.

In order to improve the applicability and user experience of the CBS3-LandGen model in actual landscape design, this study designed and introduced a user control mechanism (Fig 5), which allows designers to dynamically adjust and customize the model output during the generation process. This mechanism is based on the latent vector and style control vector of the StyleGAN3 generation optimization module. Designers can flexibly control the key design elements such as texture, color, shape and spatial layout of the generated image by adjusting these parameters, thereby achieving refined intervention in the landscape design style and functional layout.

As shown in Fig 5, the user control mechanism provides an interactive adjustment interface, allowing designers to intuitively observe the immediate impact of adjusting parameters on the generation results. By adjusting the key dimensions in the latent vector, designers can influence the theme and style changes of the overall design; while the style control vector allows for more refined adjustments to the detail level of the image, such as vegetation density, road direction or water body morphology. This interactive process, combined with parameter sensitivity analysis, allows users to clearly understand the specific contribution of each parameter to the design effect, avoid blind parameter adjustment, and improve adjustment efficiency and design accuracy. In addition, the user control mechanism supports the "human-in-the-loop" design process, that is, the designer’s adjustments not only affect the current generation results, but also feed back to the model for iterative optimization, promoting the model to gradually adapt to user preferences and generate more personalized design solutions that meet the needs. This closed-loop feedback mechanism enhances the model’s adaptive ability and improves the diversity and practicality of design solutions.

By introducing the user control mechanism, CBS3-LandGen not only achieves high-quality automated landscape design generation, but also meets the designer’s needs for transparency and autonomous adjustment of the generation process, greatly enriching the model’s application scenarios and interactive experience, and providing a more practical solution for the field of intelligent landscape design.

## Expertment

### Experimental environment

This paper conducted comprehensive experiments in a standard deep learning experimental environment. The hardware and software configurations used in the experiments are capable of handling large-scale data processing and complex model training, ensuring the reliability and efficiency of the results. In this experiment, we used the high-performance NVIDIA A100 GPU to accelerate the model training process, with optimization through CUDA and cuDNN libraries to maximize training efficiency and computational performance. All experiments were conducted on a Linux operating system, using PyTorch as the deep learning framework to ensure the efficient execution of tasks such as convolution operations, generation optimization, and multimodal data fusion. Table 1 lists the main hardware configuration, software environment, and deep learning framework used in the experiments.

**Table 1:** Experimental Environment Configuration and Hardware-Software Specifications: Computing Platform Configuration for Evaluating the Performance and Efficiency of CBS3-LandGen Model in Landscape Design Generation Tasks.

Hardware Configuration	Specifications	Software Configuration	Version
GPU	4 x NVIDIA A100 40GB	Deep Learning Framework	PyTorch 1.12.0
CPU	AMD EPYC 7F72 24 cores 2.4 GHz	CUDA Version	CUDA 11.3
Memory	256GB DDR4 RAM	cuDNN Version	cuDNN 8.3.1
Storage	2TB NVMe SSD	Python Version	Python 3.8.10
Operating System	Ubuntu 20.04 LTS	Libraries	numpy, pandas, matplotlib, scikit-learn

The hardware configuration provided strong computational power for the experiment, allowing for the efficient handling of multiple tasks and parallel computations. The high performance of the NVIDIA A100 GPUs enabled the training of deep network models (such as ConvNeXt, BART, and StyleGAN3) to be completed in a short period while ensuring stability and efficiency when processing large-scale landscape design datasets. The 256GB of memory ensured smooth data loading and model training, avoiding potential computational delays due to memory bottlenecks. In terms of storage, high-speed NVMe SSDs significantly improved data read and write speeds, optimizing data flow efficiency during the training process.

On the software side, PyTorch was chosen as the deep learning framework, as it provides comprehensive support for complex structures such as convolutional networks, generative adversarial networks, and Transformers in the model. With the acceleration provided by CUDA and cuDNN, the model’s performance on the GPU was significantly improved, further speeding up the training and inference processes.

Each training phase, especially during the pre-training and fine-tuning processes of the deep learning model, is carried out under this rigid configuration to ensure that large-scale datasets and multimodal tasks can be successfully completed. On the DeepGlobe dataset, the training time for the entire model is approximately 7 days, and 4 GPUs are used for parallel computing. For the COCO dataset, the training time is slightly longer, about 9 days. The specific training time data are shown in Table 2.

**Table 2:** Training Time and Computational Resource Configuration of the CBS3-LandGen Model on Different Datasets.

Dataset	Training Time (days)	Number of GPUs	Memory Configuration	Storage Configuration
DeepGlobe	7	4	256GB DDR4 RAM	2TB NVMe SSD
COCO	9	4	256GB DDR4 RAM	2TB NVMe SSD

### Datasets and preprocessing

In this paper, we selected two publicly available datasets for the experiments: the DeepGlobe Land Cover Classification Dataset and the COCO Dataset. These two datasets contain rich image and text data, which can effectively support tasks in multimodal learning, including image feature extraction, text understanding and generation, and multimodal data fusion.

**Fig 6:**
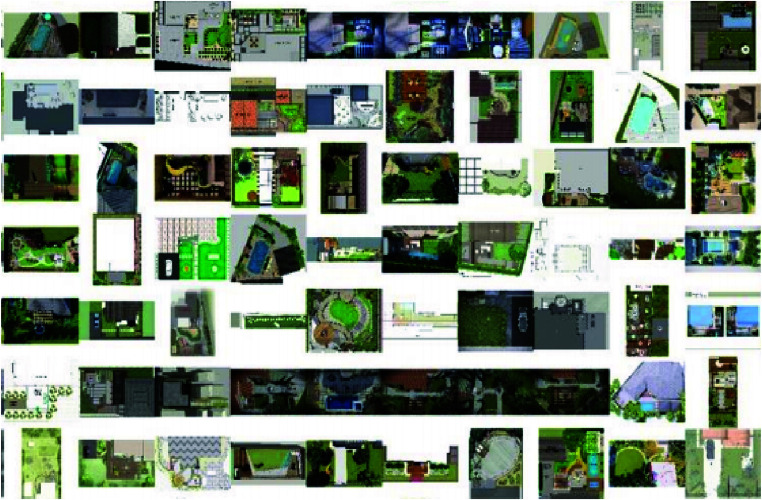
Landscape Design Examples: Displays various landscape design layouts, including parks, courtyards, urban areas, and other types of designs, showcasing diverse functional area planning and aesthetic effects. Data sourced from the publicly available DeepGlobe Land Cover Classification Dataset.

**Fig 7:**
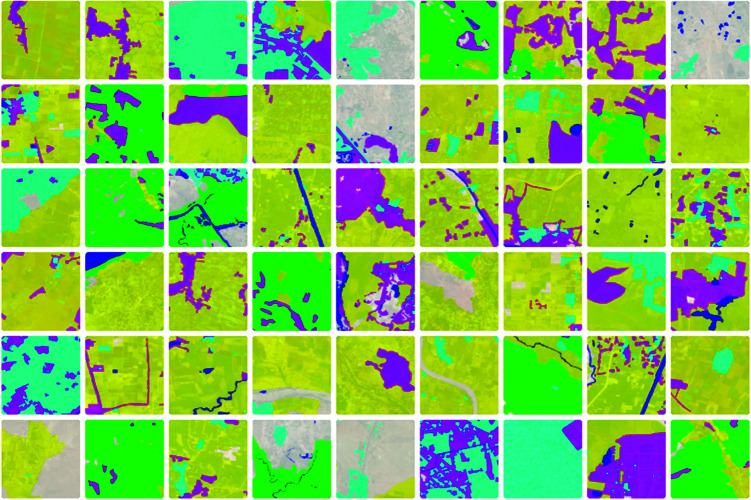
DeepGlobe Land Cover Classification Examples: Shows satellite images processed by classification algorithms, with land cover types such as agricultural land, forests, urban areas, and water bodies marked and differentiated using various colors. Data sourced from the publicly available DeepGlobe Land Cover Classification Dataset.

The DeepGlobe Land Cover Classification Dataset provides high-resolution remote sensing images covering various land types, such as urban areas, forests, and agricultural fields. While the dataset is primarily used for land cover classification tasks, it provides valuable geographical information and spatial structure for the image modality in landscape design generation [45]. The COCO Dataset, on the other hand, contains a large amount of object recognition, segmentation, and caption data, providing rich functional requirements, design descriptions, and image-related information for the text modality [46]. The reason for using these datasets lies in its open access and availability. Table 3 provides relevant information for these datasets, Figs 6 and 7 show some example data. These two datasets can be downloaded for free online: https://datasetninja.com/deepglobe and https://paperswithcode.com/dataset/coco.

**Table 3:** Basic Information of the DeepGlobe Land Cover Classification Dataset and COCO Dataset: Displaying dataset type, size, description, and their application in landscape design generation tasks.

Dataset Name	Type	Size	Description	Use
DeepGlobe Land Cover Classification Dataset	Remote Sensing Images	1,850 images	High-resolution satellite images for land cover classification, including urban, forest, and agricultural types	Provides geographical information and spatial structure for the image modality
COCO Dataset	Images and Text Descriptions	330,000 images	Contains multi-class object recognition, segmentation, and captions, including landscape elements, functional requirements, etc.	Provides design descriptions and requirements for the text modality

These datasets provide diverse inputs for model training, ensuring effective multimodal fusion of image and text data. In the data preprocessing phase, we performed cleaning and normalization of both image and text data. For the image data, we first applied normalization, adjusting the pixel values to a consistent range (typically [0, 1] or [-1, 1]) to ensure consistent scale and color across different images. Additionally, data augmentation techniques (e.g., rotation, translation, scaling) were applied to increase the diversity of the training data and enhance model robustness. To reduce noise in the images, we also applied denoising techniques such as Gaussian blur. For the text data, we first removed irrelevant words and punctuation, followed by tokenization to convert the text into word vectors. Then, we used a pre-trained word embedding model (BERT) to convert the words into vectors for further processing in the BART model. These preprocessing steps ensure high-quality and consistent data, enabling the model to effectively learn the associations between multimodal data.

### Model parameter settings

To ensure the effectiveness and stability of the CBS3-LandGen Model in landscape design generation tasks, this experiment carefully set hyperparameters for each module (ConvNeXt, BART, StyleGAN3). Table 4 presents the hyperparameter configurations for each module, including learning rate, batch size, and training epochs.

**Table 4:** Hyperparameter Settings for Each Module of the CBS3-LandGen Model: Including Learning Rate, Batch Size, and Training Epochs for ConvNeXt, BART, and StyleGAN3.

Module	Hyperparameter	Setting Value	Description
ConvNeXt	Learning Rate	0.0001	A smaller learning rate to prevent gradient explosion and ensure stable training.
	Batch Size	32	A smaller batch size to fit memory and enhance training stability.
	Training Epochs	50	After experimental tuning, 50 epochs yield optimal training results.
BART	Learning Rate	0.0003	A smaller learning rate for better context capture from text data.
	Batch Size	16	Suitable batch size for text data processing, ensuring memory efficiency.
	Training Epochs	40	Set according to text data complexity to ensure sufficient model training.
StyleGAN3	Learning Rate	0.00005	A smaller learning rate is required for generative models to avoid instability during training.
	Batch Size	8	Smaller batch size suitable for training the generative network, preventing memory overflow.
	Training Epochs	100	A longer training time for the style generation network is required to obtain high-quality images.

The learning rate of the ConvNeXt module is set to 0.0001. This value of the learning rate is an optimal value obtained through experiments. With a relatively small learning rate, the network can better avoid the problem of gradient explosion and, at the same time, ensure the stability of the model during the training process. The batch size is set to 32. Considering the limitations of the video memory and the training efficiency, this choice can ensure the memory usage while also improving the training stability. After multiple experimental verifications, a training epoch number of 50 can ensure that the model is fully trained and avoid overfitting.

The learning rate of the BART module is set to 0.0003, the batch size is 16, and the number of training epochs is 40. These hyperparameters are selected based on the complexity of capturing context information when BART processes text data. A smaller learning rate helps to better capture the long-range dependencies in the text, and an appropriate batch size and number of training epochs can balance the memory usage efficiency and the full training of the model.

For the StyleGAN3 module, the learning rate is set to 0.00005, and this choice aims to avoid instability during the generation process. The batch size is 8, and a smaller batch size helps to reduce the video memory occupation and improve the training stability. The number of training epochs is set to 100. This choice is based on the fact that the generative network requires a relatively long time to achieve high-quality image generation, especially for style adjustment and image optimization, which require a long time of training.

In all modules, we use the Adam optimizer with learning rate decay to ensure that the learning rate gradually decreases during the training process, thereby accelerating convergence and preventing overfitting. After every 10 training epochs, the learning rate will be reduced to 0.1 times its original value, ensuring that the model can refine its learning and converge stably in the later stages. In addition, an early stopping strategy is introduced during the training process. When the performance on the validation set does not significantly improve for 10 consecutive epochs, we terminate the training in advance to avoid continuing the training when the model is in a state of overfitting.

### Metrics

In the experiments of this paper, five main evaluation metrics were chosen to comprehensively assess the performance of the CBS3-LandGen Model in landscape design generation tasks. These metrics cover the quality of generated images (FID, IS), text generation quality (BLEU, ROUGE), and the consistency between images and text (CLIP Score), which can effectively reflect the overall performance of the multimodal generation model.

**FID** (Fréchet Inception Distance) is a commonly used metric to measure the similarity between generated and real images. It evaluates the image quality by calculating the Fréchet distance between the feature distributions of real and generated images.

$$\text{FID}(p,q)={\left\|\mu_{p}-\mu_{q}\right\|}^{2}+\text{Tr}\left(\Sigma_{p}+\Sigma_{q}-2(\Sigma_{p}\Sigma_{q}{)}^{1/2}\right)$$ (13)

where $\mu_{p}$ and $\mu_{q}$ are the mean vectors of real and generated images, and $\Sigma_{p}$ and $\Sigma_{q}$ are their respective covariance matrices. A smaller FID value indicates higher similarity between generated and real images, implying better image quality.

**IS** (Inception Score) evaluates the quality and diversity of generated images, commonly used for generative model evaluation. It measures the classification confidence and diversity of generated images.

$$\text{IS}(p)=\exp\left({𝔼}_{x}\left[D_{KL}(p(y|x)||p(y))\right]\right)$$ (14)

where p(y|x) is the conditional probability distribution of image label *y* given image *x*, *p*(*y*) is the marginal distribution of labels, and *D*_*KL*_ is the Kullback-Leibler divergence, representing the diversity and clarity of generated images. A higher IS value indicates better image quality and diversity.

**BLEU** (Bilingual Evaluation Understudy) is primarily used to evaluate the similarity between generated text and reference text, especially in machine translation tasks. In this task, BLEU is used to measure the consistency between generated text descriptions and design requirements.

$$\text{BLEU}(p)=\exp\left(\min(0,1-\frac{p_{n}}{p_{r}})\right)$$ (15)

where *p*_*n*_ is the n-gram match between generated text and reference text, and *p*_*r*_ is the frequency of n-grams in the reference text. A higher BLEU value indicates higher overlap between generated and reference text, implying better text quality.

**ROUGE** (Recall-Oriented Understudy for Gisting Evaluation) is a commonly used metric for evaluating text generation quality, particularly suitable for measuring the overlap between generated and reference texts.

$$\text{ROUGE-L}=\frac{\sum_{i=1}^{n}\text{LCS}(r_{i},h_{i})}{\sum_{i=1}^{n}|h_{i}|}$$ (16)

where $\text{LCS}(r_{i},h_{i})$ denotes the length of the longest common subsequence between the reference text *r*_*i*_ and the generated text *h*_*i*_, and |*h*_*i*_| is the length of the generated text. A higher ROUGE value indicates better similarity between generated and reference text, implying better text quality.

**CLIP Score** measures the semantic consistency between images and text by calculating the similarity between the feature vectors of images and text.

$$\text{CLIP Score}(x,t)=\frac{f_{clip}(x)\cdot f_{clip}(t)}{\left\|f_{clip}(x)\|\|f_{clip}(t)\right\|}$$ (17)

where *f*_*clip*_(*x*) and *f*_*clip*_(*t*) are the CLIP feature vectors of image *x* and text *t*, and ‖·‖ denotes the L2 norm of a vector. A higher CLIP Score indicates better semantic consistency between the image and text, making the generated image and text more coherent.

By combining these metrics, we can comprehensively evaluate the performance of the CBS3-LandGen Model in landscape design generation tasks and further optimize the model to achieve higher-quality generation results.

### Comparative experiment

In the comparative experiments, we selected several typical models for comparison, including unimodal generation models (Pix2Pix, CycleGAN, DeepLabV3+, etc.), multimodal models such as CLIP, VILBERT, and United, as well as improved models based on Generative Adversarial Networks (GANs) like AttnGAN. These models cover tasks such as image generation and image-text fusion. By comparing with these models, we are able to analyze the strengths and weaknesses of the CBS3-LandGen Model from multiple dimensions, including generated image quality, text generation consistency, and semantic fusion between images and text. The experimental results are shown in Table 5.

**Table 5:** Performance Comparison of CBS3-LandGen Model and Other Baseline Models on DeepGlobe and COCO Datasets.

Models	DeepGlobe Land Cover Classification Dataset					COCO Dataset				
	FID	IS	BLEU	ROUGE	CLIP Score	FID	IS	BLEU	ROUGE	CLIP Score
Pix2Pix [47]	45.2	3.4	0.28	0.42	0.75	50.1	3.1	0.30	0.44	0.73
CycleGAN [48]	47.3	3.2	0.26	0.40	0.74	52.0	2.9	0.28	0.41	0.71
DeepLabV3+ [49]	42.3	3.3	0.29	0.41	0.76	46.7	3.0	0.31	0.43	0.74
CLIP [50]	30.1	3.8	0.34	0.47	0.83	35.2	3.6	0.36	0.50	0.81
AttnGAN [51]	40.5	3.6	0.30	0.43	0.78	43.7	3.3	0.32	0.46	0.76
VILBERT [52]	38.9	3.7	0.32	0.45	0.80	42.5	3.4	0.33	0.48	0.78
United [53]	39.6	3.5	0.31	0.44	0.79	44.1	3.2	0.32	0.47	0.77
**CBS3-LandGen**	**25.5**	**4.3**	**0.42**	**0.55**	**0.88**	**30.2**	**4.0**	**0.45**	**0.58**	**0.86**

From the Table 5, it can be seen that CBS3-LandGen outperforms all other models on every evaluation metric. Taking the results on the DeepGlobe dataset as an example, on the FID metric, CBS3-LandGen achieves a value of 25.5, significantly lower than that of other comparison models, demonstrating that its generated images are of superior quality. A low FID value indicates that the generated images are closer to the real images, with higher authenticity and quality, proving the model’s excellence in image generation. On the IS metric, CBS3-LandGen scores 4.3, ranking higher than all other models, significantly surpassing the scores of the other models. A high IS value suggests that the generated images not only have higher quality but also exhibit better diversity, indicating that this model can preserve details and present rich variation and creativity, which is particularly important for the diverse requirements of landscape design tasks.

In text generation evaluation metrics, BLEU and ROUGE, CBS3-LandGen also performs optimally, achieving scores of 0.42 and 0.55, respectively, far surpassing other models. These two metrics indicate that the text descriptions generated by this model are the most similar to and have the highest overlap with the reference texts, demonstrating the best quality in text generation. Compared to traditional image generation models, this highlights the advantages of CBS3-LandGen in the effective fusion of image and text, allowing it to generate text descriptions that are both aligned with design requirements and highly consistent. In the CLIP Score, CBS3-LandGen scores 0.88, the highest among all models. This result shows that this model far exceeds other models in terms of semantic consistency between image and text, achieving the best correlation and consistency between the two. Additionally, experiments on the COCO dataset also show equally excellent performance.

**Fig 8:**
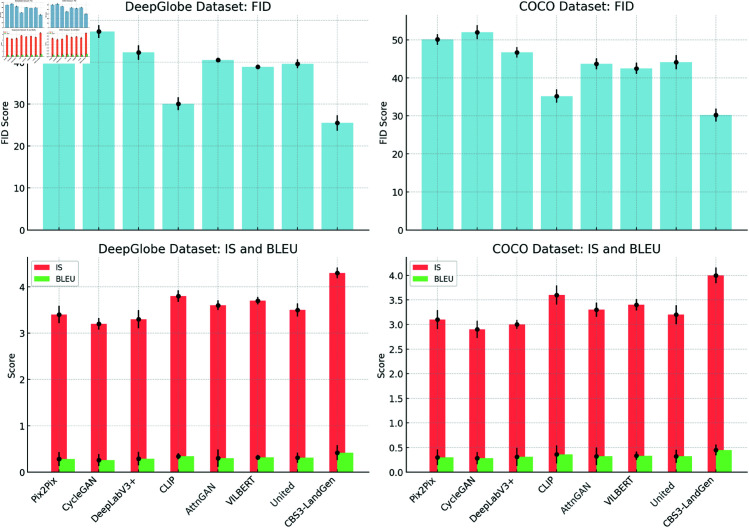
Performance Comparison Visualization of CBS3-LandGen Model and Other Baseline Models on DeepGlobe and COCO Datasets: Including FID, IS, and BLEU Metrics, with Error Bars.

Fig 8 visualizes the results of the comparative experiments, showcasing the comparison of FID, IS, and BLEU metrics. From the figure, it can be seen that CBS3-LandGen significantly outperforms other models on these three key metrics, particularly on FID and IS, demonstrating a clear advantage in image generation quality and diversity. The error bars in the figure further confirm the stability of CBS3-LandGen’s generation performance, with its error range being much smaller than that of other models. This indicates that the model not only generates high-quality outputs but also exhibits strong robustness. These results validate CBS3-LandGen’s exceptional performance in image generation and text consistency, and highlight its consistency and advantages across different datasets."

We believe that the reason CBS3-LandGen achieves the best performance across all evaluation metrics is due to its multimodal generation architecture and innovative module design. First, the ConvNeXt module fully utilizes the advantages of deep convolutional neural networks in processing image modality, extracting deep features of images and generating higher-quality and more diverse images. The BART module effectively processes the text modality, demonstrating excellent capability in understanding and generating text, and can produce design descriptions that meet practical requirements. The StyleGAN3 module optimizes the generation process, enhancing the aesthetic effect and functionality of the images, ensuring a balance between multiple design objectives in the generated landscape design images. Through the organic integration of these modules, CBS3-LandGen achieves a high level of synergy between image and text, ensuring that the generated results achieve the best outcomes in both visual quality and textual consistency. The close collaboration between the modules, combined with advanced deep learning techniques, addresses the shortcomings of traditional generation models in image and text fusion, providing a novel, multimodal, and efficient solution for landscape design generation.

### Ablation experiment

For the ablation experiments, we gradually removed individual modules from the CBS3-LandGen model to assess their impact on overall performance. The experimental results are shown in Table 6.

**Table 6:** Performance Comparison of CBS3-LandGen Model and Ablated Models on DeepGlobe and COCO Datasets: Impact of Module Removal on Generation Quality, Text Consistency, and Multi-Modal Fusion.

Models	DeepGlobe Land Cover Classification Dataset					COCO Dataset				
	FID	IS	BLEU	ROUGE	CLIP Score	FID	IS	BLEU	ROUGE	CLIP Score
Full CBS3-LandGen	**25.5**	**4.3**	**0.42**	**0.55**	**0.88**	**30.2**	**4.0**	**0.45**	**0.58**	**0.86**
without ConvNeXt	35.2	3.7	0.36	0.50	0.82	40.1	3.5	0.38	0.52	0.80
without BART	38.4	3.5	0.33	0.47	0.80	43.2	3.2	0.35	0.49	0.77
without StyleGAN3	40.1	3.3	0.30	0.44	0.78	44.0	3.1	0.32	0.47	0.75

From Table 6, it is clear that the CBS3-LandGen model outperforms all ablation models across all evaluation metrics, particularly in FID, IS, and CLIP Score. Removing any single module results in a significant performance drop, proving the critical role each module plays in the overall performance.

When the ConvNeXt module is removed, the FID value increases to 35.2, the IS value drops to 3.7, and the CLIP Score decreases to 0.82. These changes indicate that the ConvNeXt module is crucial for image feature extraction and generation quality. After removing this module, the quality and diversity of the generated images decrease, and the semantic consistency between the image and text is also impacted.

After removing the BART module, the BLEU and ROUGE scores decrease to 0.33 and 0.47, respectively, and the CLIP Score further drops to 0.80. This change suggests that the BART module plays an important role in text generation and the consistency between text and images. Without this module, the quality of generated text and its alignment with the image significantly decline.

When the StyleGAN3 module is removed, the quality of the generated images decreases significantly, with the FID value rising to 40.1, the IS value dropping to 3.3, and the CLIP Score falling to 0.78. This indicates that the StyleGAN3 module plays a vital role in optimizing the aesthetic quality, functionality of generated images, and their consistency with the text.

**Fig 9:**
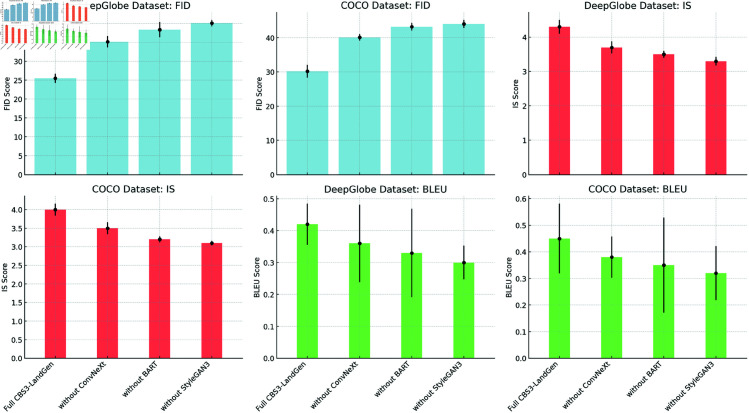
Visualization of the ablation experiment results for the CBS3-LandGen model: Impact of gradually removing each module on performance (FID, IS, and BLEU) on the DeepGlobe and COCO datasets, with error bars indicated.

Fig 9 visualizes the results of the ablation experiments for the CBS3-LandGen model using key metrics such as FID, IS, and BLEU. From the error bars in the figure, it is clear that removing the ConvNeXt, BART, or StyleGAN3 modules leads to a significant drop in the model’s performance in terms of generation quality, text consistency, and multi-modal fusion.

This indicates that each module contributes significantly to the final model performance, and removing any one module negatively affects the generation results. This validates the critical role each module plays in the overall performance of the CBS3-LandGen model. The synergy between the modules enables CBS3-LandGen to exhibit unparalleled advantages in image generation quality, text generation consistency, and the fusion of image and text.

### Results

In the experiment, we evaluated the performance of the CBS3-LandGen model when facing different levels of noise and mismatched image-text pairs (Table 7) by gradually increasing the proportions of noise and mismatched image-text pairs (20% and 40% respectively), as well as combining the two (20% noise and 40% mismatch).

**Table 7:** Sensitivity Analysis of CBS3-LandGen Model on Noisy and Mismatched Image-Text Pairs: Impact on Generation Quality and Cross-Modal Alignment.

Models	DeepGlobe Land Cover Classification Dataset					COCO Dataset				
	FID	IS	BLEU	ROUGE	CLIP Score	FID	IS	BLEU	ROUGE	CLIP Score
Full CBS3-LandGen	**25.5**	**4.3**	**0.42**	**0.55**	**0.88**	**30.2**	**4.0**	**0.45**	**0.58**	**0.86**
20% Noisy	32.1	3.9	0.39	0.50	0.85	36.7	3.6	0.41	0.53	0.82
40% Noisy	38.4	3.6	0.35	0.48	0.81	42.1	3.3	0.37	0.51	0.78
20% Mismatched	34.5	3.8	0.38	0.49	0.83	38.9	3.5	0.40	0.52	0.80
40% Mismatched	41.2	3.4	0.33	0.46	0.79	45.4	3.1	0.34	0.48	0.75
20% Noisy & Mismatched	43.7	3.3	0.30	0.44	0.77	47.3	3.0	0.32	0.46	0.72

In terms of the Frechet Inception Distance (FID) value, as the noise and mismatch increase, the generation quality of the model significantly deteriorates. For example, on the DeepGlobe dataset, as the noise proportion increases from 0% to 40%, the FID value rises from 25.5 to 41.2, showing a clear decline in quality. Similarly, the FID value of the COCO dataset also exhibits a similar trend, increasing from 30.2 to 47.3. This indicates that when there is a large amount of noise or mismatched image-text pairs in the input data, the authenticity and quality of the images generated by the model are greatly affected.

On the Inception Score (IS), the generation diversity of the model is also impacted. When the proportion of noise or mismatch increases, the IS value gradually decreases, reflecting that the diversity and innovativeness of the model during generation are restricted. For instance, on the DeepGlobe dataset, the IS value drops from the original 4.3 to 3.3 (when the noise and mismatched pairs reach 20% and 40% respectively), which indicates that the diversity of the generated images has decreased, and the model may become more conservative, resulting in more homogeneous generated design plans.

In terms of the BLEU and ROUGE metrics, as the proportion of mismatched image-text pairs increases, the quality of the text generated by the model also declines. In the scores of BLEU and ROUGE, as the proportions of noise and mismatched image-text pairs increase, the scores drop from the original 0.42 and 0.55 to 0.30 and 0.44 respectively. This shows that the similarity and overlap between the generated text descriptions and the reference texts are weakened. Especially when the alignment of image and text is inaccurate, the text generation quality of the model is significantly affected.

As an indicator for measuring the semantic consistency between images and texts, the CLIP Score also shows a similar downward trend. As the proportions of noise and mismatch increase, the CLIP Score drops from the original 0.88 to 0.77, indicating that when facing inconsistent image-text pairings, the semantic consistency between the images and texts generated by the model decreases, and the matching degree between the generated images and texts significantly reduces.

These results suggest that the CBS3-LandGen model shows a high level of sensitivity when facing noisy and mismatched image-text pairs. Although the model can generate high-quality landscape design images and maintain good alignment between images and texts under normal circumstances, when the quality of the input data decreases, the generation effect of the model is significantly impaired.

Table 8 shows the sensitivity analysis results of the StyleGAN3 generation module in the CBS3-LandGen model to the key parameter perturbations of the latent vector and style control vector. As the parameter perturbation amplitude gradually increases from no perturbation to ±60%, the FID value of the generated image shows a gradual upward trend, indicating that the image quality has declined compared with the baseline, but is still within a reasonable range; at the same time, the IS value decreases accordingly, reflecting that the diversity and innovation of the generated images are slightly weakened. Visual observation shows that parameter adjustment can significantly affect the texture details, color style, spatial layout and shape structure of the image, fully demonstrating the model’s ability to adjust design style and details. Overall, this experiment verifies that the StyleGAN3 module in CBS3-LandGen has good parameter controllability and stability, provides designers with effective interactive control methods, and enhances the practical application potential of the model.

**Table 8:** Sensitivity Analysis of StyleGAN3 Parameters in CBS3-LandGen Model: Impact on Generation Quality and Diversity.

Parameter Perturbation	FID	IS
No Perturbation (Baseline)	25.5	4.3
±20% Adjustment	27.8	4.1
±40% Adjustment	30.6	3.9
±60% Adjustment	35.2	3.6

**Fig 10:**
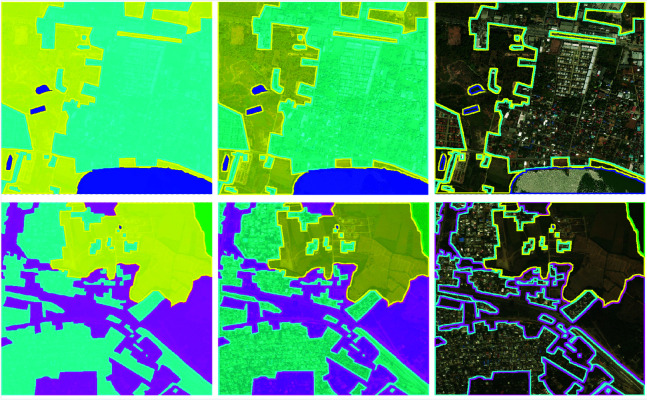
CBS3-LandGen Model’s Landscape Design Generation Experimental Results in Different Regions: Displays the model’s performance in multiple areas (e.g., urban, suburban, and rural). The land cover types in each area (such as agricultural land, urban land, water bodies, etc.) are marked using different colors, showcasing the comparison between the generated landscape designs and ground truth data.

Fig 10 shows the CBS3-LandGen model’s landscape design generation results for multiple urban and rural areas. As can be seen from the figure, the model performs differently in different areas. For urban areas, the model can effectively identify and generate complex buildings, roads, and other infrastructure areas (marked in yellow and green). For rural and suburban areas, the model focuses more on the generation of natural landscapes, such as agricultural land and water bodies (represented in blue and pink). The boundaries of each image are marked in yellow, indicating the correspondence between the model-generated areas and the ground truth data. Through this visualization, the results show that the CBS3-LandGen model can achieve accurate landscape design generation and effectively integrate urban and natural landscape elements when dealing with diverse land cover types. The model’s multimodal processing capabilities enable it to generate designs that meet ecological, functional, and aesthetic requirements in different types of geographic areas, verifying its wide applicability in the automated generation of landscape designs.

**Fig 11:**
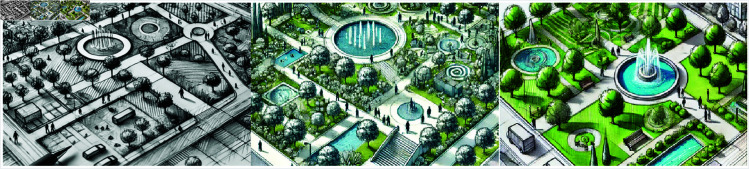
Landscape Design Schemes Generated by CBS3-LandGen Model: Displays different stages of the park landscape design sketches, progressively from black-and-white hand-drawn sketches to color-detailed designs, highlighting the layout of green spaces, water features, pathways, and leisure areas.

Fig 11 shows the CBS3-LandGen model’s process in landscape design generation, from preliminary black and white sketches to gradually refined color designs. Through this step-by-step optimization process, the model is able to gradually generate more detailed and vivid designs, starting with simple functional area layouts (such as trails, water features, and trees). The images of each stage clearly show how the model generates a landscape design that meets ecological, functional, and aesthetic requirements based on input data (such as topographic maps, functional requirements, and text descriptions). In the preliminary design stage (left), the model output a simple park layout with basic trails and water features, but lacks details and colors. In the optimization stage (center), the model adds more details, such as the refinement of water features, the rational layout of vegetation distribution, and more vivid colors and shadow effects. The design at this stage pays more attention to the use of space and enhances the functionality and aesthetic performance of different areas. The final design (right) shows a refined landscape design scheme that not only includes abundant green space, water features, and recreational areas, but also enhances the ecological benefits and functionality of the landscape, such as the rational layout of seats and rest areas to ensure user experience.

Through this design generation process, the model demonstrates its powerful image generation capabilities and multimodal data processing abilities, accurately converting the input design requirements into practical and executable landscape design solutions. These results show that the CBS3-LandGen model can handle complex landscape design tasks while providing high-quality designs that meet the requirements.

### Discussion

In the experiments, although the CBS3-LandGen model has demonstrated strong capabilities in various landscape design tasks, it still faces some challenges, particularly in terms of instability in generation quality. While most of the design results align with expectations, the model can sometimes produce overly simplified or insufficiently detailed outputs, especially in complex areas, leading to unstable image quality. This issue is particularly noticeable when handling complex spatial layouts and multimodal input data, where the generated results sometimes lack fine textures and realism. Although this issue can be alleviated to some extent by optimizing the model structure and diversifying the training dataset, the existing datasets (such as COCO and DeepGlobe) have not fully reflected the input data in actual landscape design, especially in terms of the utilization of professional landscape design documents or urban planning data. Therefore, future research will focus on exploring the introduction of more practical and specialized datasets, such as data from actual landscape design projects, urban planning documents, and environmental sensor data. This will help improve the practical application value and accuracy of the model, and ensure that in more complex and diverse scenarios, the model can generate more stable and high-quality design plans.

Additionally, training time is another significant challenge the model faces. Since the CBS3-LandGen model involves the collaborative work of multiple modules, such as image processing, text understanding, and generation optimization, these complex operations require substantial computational resources and training time. Especially during the training process with large-scale datasets, the model’s training cycle is long, creating a considerable computational burden. This not only affects the efficiency of the experiments but also increases the difficulty of deploying and applying the model. To address this issue, future research will focus on optimizing training efficiency and reducing computational costs. We plan to explore some efficient training strategies, such as model distillation, distributed training, mixed-precision training, and other methods, in order to reduce the training time and enhance the practicality and real-time performance of the model.

## Conclusion

With the acceleration of the global urbanization process, landscape design is confronted with increasingly complex challenges. Traditional manual design methods are gradually failing to meet the requirements for rapid, efficient, and sustainable design. This paper proposes an intelligent landscape design generation model, CBS3-LandGen, based on multimodal deep learning. By integrating image data, text data, and generation optimization techniques, this model achieves efficient multimodal data fusion and is capable of generating landscape plans that meet ecological, functional, and aesthetic requirements under multiple design constraints. Experimental results show that CBS3-LandGen outperforms existing methods in terms of image generation quality, text consistency, and the fusion of images and text, demonstrating great potential for intelligent landscape design.

Nevertheless, the generation quality of CBS3-LandGen still exhibits instability in some complex scenarios, especially in the performance of details, where there is still room for improvement. In addition, the long training time and computational resources required during the model training process, particularly when training on large-scale datasets, also limit its efficiency in practical applications.

Future research will focus on addressing these shortcomings to improve the generation quality and training efficiency. We plan to introduce more advanced generative network architectures and optimization algorithms to reduce the training time and enhance the consistency of the results. At the same time, we will further expand the application scope of the model by incorporating more modalities of data, such as environmental sensor data, real-time urban planning data, etc., to enhance its adaptability and generalization ability. By improving the robustness of the model to cope with more complex design requirements, it will contribute to the model’s performance in practical applications. We will also accelerate the training process through parallel computing and efficient optimization algorithms to improve the real-time application ability of the model. Finally, future research will focus on exploring the application of the model in practical scenarios such as urban planning and ecological protection, promoting the digitalization and intelligentization development in the field of landscape design, and providing innovative solutions for the construction of sustainable cities.

## References

[1] Chen X. Environmental landscape design and planning system based on computer vision and deep learning. J Intell Syst. 2023;32(1):20220092.

[2] Zeng X, Zhong Y, Yang L, Wei J, Tang X. Analysis of forest landscape preferences and emotional features of Chinese forest recreationists based on deep learning of geotagged photos. Forests. 2022;13(6):892.

[3] Jia F. Neural network model of urban landscape design based on multi-target detection. Comput Intell Neurosci. 2022;2022:9383273. doi: 10.1155/2022/9383273 35909821 PMC9325590

[4] Fan J. Design of 3D interactive landscape roaming algorithm based on deep learning technology. In: 2022 IEEE Asia-Pacific Conference on Image Processing, Electronics and Computers (IPEC). 2022. p. 1143–5. 10.1109/ipec54454.2022.9777396

[5] Stupariu MS, Cushman SA, Ple¸soianu AI, P˘atru-Stupariu I, Fuerst C. Machine learning in landscape ecological analysis: a review of recent approaches. Landscape Ecol. 2022;37(5):1227–50.

[6] Chen R, Zhao J, Yao X, He Y, Li Y, Lian Z, Li H. Enhancing urban landscape design: a GAN-based approach for rapid color rendering of park sketches. Land. 2024;13(2):254.

[7] Shan P, Sun W. Research on landscape design system based on 3D virtual reality and image processing technology. Ecol Inform. 2021;63:101287. doi: 10.1016/j.ecoinf.2021.101287

[8] Zhang D, Huda S, Songhori E, Prabhu K, Le Q, Goldie A, et al. A full-stack search technique for domain optimized deep learning accelerators. In: Proceedings of the 27th ACM International Conference on Architectural Support for Programming Languages and Operating Systems. 2022. p. 27–42. 10.1145/3503222.3507767

[9] Maxwell AE, Warner TA, Guillén LA. Accuracy assessment in convolutional neural network-based deep learning remote sensing studies—Part 2: recommendations and best practices. Remote Sens. 2021;13(13):2591.

[10] Kido D, Fukuda T, Yabuki N. Assessing future landscapes using enhanced mixed reality with semantic segmentation by deep learning. Adv Eng Inf. 2021;48:101281. doi: 10.1016/j.aei.2021.101281

[11] Havinga I, Marcos D, Bogaart PW, Hein L, Tuia D. Social media and deep learning capture the aesthetic quality of the landscape. Sci Rep. 2021;11(1):20000. doi: 10.1038/s41598-021-99282-0 34625594 PMC8501120

[12] Zhang L, Liu J, Wei Y, An D, Ning X. Self-supervised learning-based multi-source spectral fusion for fruit quality evaluation: a case study in mango fruit ripeness prediction. Inf Fusion. 2025;117:102814. doi: 10.1016/j.inffus.2024.102814

[13] Huang J, Yu X, An D, Ning X, Liu J, Tiwari P. Uniformity and deformation: a benchmark for multi-fish real-time tracking in the farming. Exp Syst Appl. 2025;264:125653. doi: 10.1016/j.eswa.2024.125653

[14] Taşcı B. Attention deep feature extraction from brain MRIs in explainable mode: DGXAINet. Diagnostics (Basel). 2023;13(5):859. doi: 10.3390/diagnostics13050859 36900004 PMC10000758

[15] Zhang H, Yu L, Wang G, Tian S, Yu Z, Li W, et al. Cross-modal knowledge transfer for 3D point clouds via graph offset prediction. Pattern Recogn. 2025;162:111351. doi: 10.1016/j.patcog.2025.111351

[16] Yuan J, Zhang L, Kim C-S. Multimodal interaction of MU plant landscape design in marine urban based on computer vision technology. Plants (Basel). 2023;12(7):1431. doi: 10.3390/plants12071431 37050057 PMC10096508

[17] Su F. Modular information fusion model of landscape design based on genetic algorithm. In: International Conference on Multi-modal Information Analytics. 2022. p. 201–8.

[18] Yu L, Zhang X, Zhong Z, Lai Y, Zhang H, Szczerbicki E. Adaptive2Former: enhancing chromosome instance segmentation with adaptive query decoder. Cybernet Syst. 2023. p. 1–9. doi: 10.1080/01969722.2023.2296249

[19] Pokhrel K, Sanin C, Sakib MdKH, Islam MR, Szczerbicki E. Improved skin disease classification with mask R-CNN and augmented dataset. Cybernet Syst. 2023:1–15. doi: 10.1080/01969722.2023.2296254

[20] Sun M, Yan S. Research on the creative design method of Dunhuang mural art based on multi-modal fusion. In: Proceedings of the 4th International Conference on Artificial Intelligence and Computer Engineering. 2023. p. 1177–83. 10.1145/3652628.3652821

[21] Wang S, Mei L, Liu R, Jiang W, Yin Z, Deng X, et al. Multi-modal fusion sensing: a comprehensive review of millimeter-wave radar and its integration with other modalities. IEEE Commun Surv Tutorials. 2024.

[22] Alyahya K, Doherty K, Akman OE, Fieldsend JE. Reduced models of gene regulatory networks: visualising multi-modal landscapes. Metaheuristics for finding multiple solutions. Springer; 2021. p. 229–58.

[23] Liu S, Hussain AS, Sun C, Shan Y. M2 UGen: multi-modal music understanding and generation with the power of large language models. arXiv preprint 2023. doi: arXiv:2311.11255

[24] Jiang F, Ma J, Webster CJ, Chiaradia AJ, Zhou Y, Zhao Z, et al. Generative urban design: a systematic review on problem formulation, design generation, and decision-making. Prog Plann. 2024;180:100795.

[25] Wu AN, Stouffs R, Biljecki F. Generative adversarial networks in the built environment: a comprehensive review of the application of GANs across data types and scales. Build Environ. 2022;223:109477.

[26] Zhou L, Zhang X, Dong K. Does digital financial innovation contribute to promoting the high-quality development of the real economy? – Mechanism analysis and spatial econometrics based on financial service efficiency. J Xi’an Univ Financ Econ. 2024;37(01):60–72.

[27] Hughes RT, Zhu L, Bednarz T. Generative adversarial networks-enabled human-artificial intelligence collaborative applications for creative and design industries: a systematic review of current approaches and trends. Front Artif Intell. 2021;4:604234. doi: 10.3389/frai.2021.604234 33997773 PMC8113684

[28] Masenya TM. Digital transformation of medical libraries. Int J E-Health Med Commun. 2024;15(1):1–13. doi: 10.4018/ijehmc.345402

[29] Goodarzi P, Ansari M, Rahimian FP, Mahdavinejad M, Park C. Incorporating sparse model machine learning in designing cultural heritage landscapes. Autom Constr. 2023;155:105058.

[30] Chen J, Stouffs R. Deciphering the noisy landscape: Architectural conceptual design space interpretation using disentangled representation learning. Comput-Aided Civ Infrast Eng. 2023;38(5):601–20.

[31] Li M, Guenier AW. ChatGPT and health communication. Int J E-Health Med Commun. 2024;15(1):1–26. doi: 10.4018/ijehmc.349980

[32] Yang C, Liu T. Social media data in urban design and landscape research: a comprehensive literature review. Land. 2022;11(10):1796.

[33] Yuan J, Zhang L, Kim C-S. Multimodal interaction of MU plant landscape design in marine urban based on computer vision technology. Plants (Basel). 2023;12(7):1431. doi: 10.3390/plants12071431 37050057 PMC10096508

[34] Yuan J, Zhang L, Kim CS. Multimodal interaction evaluation and optimization design of smart landscape devices for elderly. 2023.

[35] Harrington MCR, Bledsoe Z, Jones C, Miller J, Pring T. Designing a virtual arboretum as an immersive, multimodal, interactive, data visualization virtual field trip. MTI. 2021;5(4):18. doi: 10.3390/mti5040018

[36] Liu S, Cao S, Lu X, Peng J, Ping L, Fan X, et al. Lightweight deep learning model, ConvNeXt-U: an improved U-Net network for extracting cropland in complex landscapes from Gaofen-2 images. Sensors (Basel). 2025;25(1):261. doi: 10.3390/s25010261 39797051 PMC11723271

[37] Zhao X, Lu Y, Lin G. An integrated deep learning approach for assessing the visual qualities of built environments utilizing street view images. Eng Appl Artif Intell. 2024;130:107805.

[38] Nasios I. Enhancing kelp forest detection in remote sensing images using crowdsourced labels with mixed vision transformers and convnext segmentation models. Int J Remote Sens. 2025:1–19.

[39] Huang G, Yu Y, Lyu M, Sun D, Zeng Q, Bart D. Using Google Street View panoramas to investigate the influence of urban coastal street environment on visual walkability. Environ Res Commun. 2023;5(6):065017.

[40] Carron B, Muys B, Orshoven JV, Leinfelder H. Landscape design to meet the societal demand for ecosystem services: a perspective. CiS. 2021;9(1):28–44. doi: 10.12924/cis2021.09010028

[41] Lanjouw L, Bart J, Mourits MJE, Willems SM, van der Hout AH, Ter Elst A, et al. BRCA1/2 testing landscape in ovarian cancer: a nationwide, real-world data study. Cancers (Basel). 2024;16(9):1682. doi: 10.3390/cancers16091682 38730634 PMC11083399

[42] Wu AN, Stouffs R, Biljecki F. Generative adversarial networks in the built environment: a comprehensive review of the application of GANs across data types and scales. Build Environ. 2022;223:109477.

[43] Alibani M, Acito N, Corsini G. Sentinel-2 image generation via Stylegan3 model. In: 2023 13th Workshop on Hyperspectral Imaging and Signal Processing: Evolution in Remote Sensing (WHISPERS). 2023. p. 1–5. 10.1109/whispers61460.2023.10430833

[44] Choi J, Seo K, Ashtari A, Noh J. StyleCineGAN: landscape cinemagraph generation using a pre-trained StyleGAN. In: Proceedings of the IEEE/CVF Conference on Computer Vision and Pattern Recognition. 2024. p. 7872–81.

[45] Demir I, Koperski K, Lindenbaum D, Pang G, Huang J, Basu S, et al. Deepglobe 2018: a challenge to parse the earth through satellite images. In: Proceedings of the IEEE Conference on Computer Vision and Pattern Recognition Workshops. 2018. p. 172–81.

[46] Lin TY, Maire M, Belongie S, Hays J, Perona P, Ramanan D, et al. Microsoft coco: common objects in context. In: Computer Vision–ECCV 2014: 13th European Conference, Zurich, Switzerland, September 6-12, 2014, Proceedings, Part V 13. 2014. p. 740–55.

[47] SENEM MO, KOC¸ M, TUNC¸ AY HE, AS I˙. Using deep learning to generate front and backyards in landscape architecture. Arch Plan J. 2023;28(3):1.

[48] Gao C, Zhang J. Enhancing image fusion and quality improvement in modern landscape architecture style design using CycleGAN with attention mechanism optimized by ResNet-50. Front Built Environ. 2023;10:1334704.

[49] Cao Q, Li M, Yang G, Tao Q, Luo Y, Wang R, et al. Urban vegetation classification for unmanned aerial vehicle remote sensing combining feature engineering and improved DeepLabV3+. Forests. 2024;15(2):382. doi: 10.3390/f15020382

[50] Horwood J, Pithara C, Lorenc A, Kesten JM, Murphy M, Turner A, et al. The experience of conducting collaborative and intensive pragmatic qualitative (CLIP-Q) research to support rapid public health and healthcare innovation. Front Sociol. 2022;7:970333. doi: 10.3389/fsoc.2022.970333 36189441 PMC9520785

[51] Chen Y. Generate lanscape paintings from text to landscape painting generation and redrawing using deep learning model. In: 2022 5th International Conference on Artificial Intelligence and Big Data (ICAIBD). 2022. p. 145–9. 10.1109/icaibd55127.2022.9820385

[52] Li X, Wen C, Hu Y, Yuan Z, Zhu XX. Vision-language models in remote sensing: current progress and future trends. IEEE Geosci Remote Sens Mag. 2024.

[53] Stevens JT, Haffey CM, Coop JD, Fornwalt PJ, Yocom L, Allen CD, et al. Tamm review: postfire landscape management in frequent-fire conifer forests of the southwestern United States. Forest Ecol Manag. 2021;502:119678. doi: 10.1016/j.foreco.2021.119678

